# Supplementary material for: TastepepAI: An artificial intelligence platform for taste peptide de novo design
Source: PLoS Comput Biol. 2025 Oct 16;21(10):e1013602. doi: 10.1371/journal.pcbi.1013602 (PMC12543283; doi:10.1371/journal.pcbi.1013602)
Supplement: S1 File — (ZIP) [file pcbi.1013602.s006.zip › Supplementary Data 2/TaPep5 HPLC.doc]

**HPLC REPORT**

| Sample: | FCl-NJP93305 TaPep5 KV-6 | | | Analyzed date: | 2024-9-21 |
| --- | --- | --- | --- | --- | --- |
| Analyst: | LY Reconstitution: 1mg/0.5ml H_2_O | | | | |
| Lot. No.: | P240913-MX1172228 | | | | |
| Column: | 4.6×250mm,ChromCore 120 C18 5u | | | | |
| Solvent A | A: 0.1% Trifluoroacetic Acid in 100% Acetonitrile | | | | |
| Solvent B | B: 0.1% Trifluoroacetic Acid in 100% Water | | | | |
| Gradient: |  | A | B |  | |
|  | 0.0min | 12% | 88% |  | |
|  | 25.0min | 37% | 63% |  | |
|  | 25.1min | 100% | 0% |  | |
|  | 30.0min | Stop | |  | |
| Volume: | 10μl | | |  | |
| Wavelength: | 220nm | | |  | |
| Flow rate: | 1.0ml/min | | |  | |
| ────────────────────────────  Rank Time Conc. Area Height  ────────────────────────────  1 8.523 0.8663 162224 23316  2 8.676 97.86 18325094 1459438  3 9.030 1.272 238248 35917  ────────────────────────────  Total 100 18725566 1518671 | | | | | |
